# Launching a new journal on the Internet in an era of fake science news and predatory publishing—doing the right thing and doing the thing right

**DOI:** 10.1002/cre2.62

**Published:** 2017-02-27

**Authors:** Asbjørn Jokstad

**Affiliations:** ^1^ CEDR UK

The dissemination of new scientific discoveries in medicine has undergone a rapid transformation from exclusively printed words, to comprise graphs, photographs, and radiographs of increasing qualities. It seems obvious that elements such as 3D animation, film videos, and tomography imaging can enhance the translation of new scientific findings for both the professionals as well as the laypersons. For this reason, I share with others the conviction that the future of dissemination of new scientific discoveries will be through digital media. Moreover, open access (OA) publishing is one approach to assure egalitarian dissemination of new scientific discoveries, and in a previous editorial in this journal, I have argued why OA appear to be a logical evolutionary extension of evidence‐based medicine (Jokstad, 2015a). Hence, the background for launching *Clinical and Experimental Dental Research* within this framework.

It is exciting to launch a new OA journal from scratch. Nevertheless, there are challenges created by the burgeoning predatory publishing industry (Jokstad, 2015b). A thought‐provoking editorial appeared recently in the *Journal of the American Dental Association* with guidance for how to identify the characteristics of predatory journals in dentistry (Glick, 2016). The keywords are scam reviews, scam journals, scam science, minimal, or no text editing amongst other deficient standards for ethical publishing. Moreover, and importantly, the editor converge also on the aspects of “publish and perish” and not on the familiar “publish or perish.” Stated more bluntly, along with other senior colleagues who regularly appraise C.V.s of academicians, I share a dilemma. How do you judge credentials if a submitted publication list include papers published in predatory journals? Is the circumstance a clear sign of poor judgment or perhaps conjectured as a questionable opportunistic trait? Alternatively, is it unkind to explore Google Scholar (that also include the contents of predatory journals) to check whether the academician have indeed published in a predatory journal but decided to suppress this information in their C.V? To expound further, if this is the case, what would then be an appropriate judgement of character? I admit that any elaboration of further ethical considerations and principles is both complex and extensive and goes beyond this short editorial. However, I advise all potential candidates for a position or tenure to reflect carefully about the prospect of their career being likely positively or negatively affected in the future by submitting today their research paper to a predatory publisher.

Until recently, many stakeholders accessed a particular website to check whether an unknown journal title could be a predatory journal, that is, whether the title was registered on the so‐called Beall's list of predatory journals (www.scholarlyoa.com). The initiatives and efforts made by Mr. Beall, a librarian affiliated with the University of Colorado in the United States, have been admirable. However, the explosive inflation of new journal titles have become simply too overwhelming to continue to update these lists and the website is therefore no longer operational. (Giles, n.d.). By own account, I can tally about 50 journals in dentistry that according to my standards should qualify to be on Beall's lists based on the abundance of invitations received by email for a paper submission, referee task, or joining an editorial board. Only approximately half of these 50 titles appear amongst the 1,294 journals on the Beall's 2017 list, which reflect an amazing creativity for inventing new journal titles in dentistry. There are profuse permutations of the words “annals or journal or archives” in combination with “dental or dentistry or oral or oro‐” in combination with “care or management” in combination with “and or &” and in combination with “health or disorders.” It extremely difficult to remember the nuances of the different names, but it may help to remember that most of these journals have individual ISSNs (International Standard Serial Number) different from the established journals.

**Figure 1:**
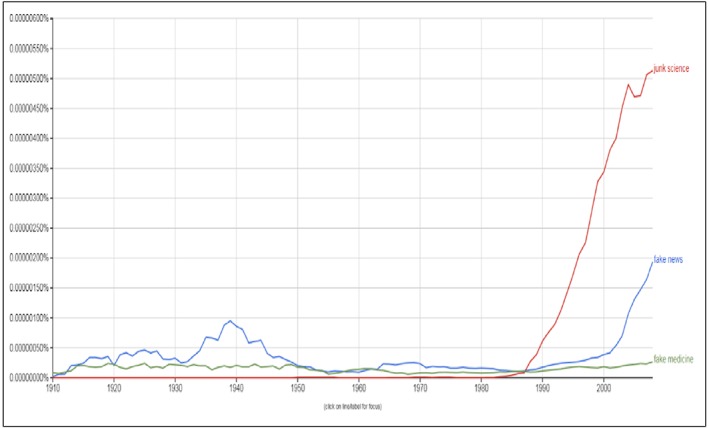
Frequency of use of the terms “junk science,” “fake news,” and “fake medicine” in the English literature in the period between 1910 and 2008 (last year of registration). Source: Google Books nomogram viewer

Over the years, questionable publications in sciences have been labelled as fake, bogus, junk, and fraudulent science. Interestingly, a new term these days seems to increase exponentially in use on the Internet, that is, the idiom “fake science news.” Fake science news appeared for the first time, according to the Google search engine, as recently as in 1995, and was used infrequently until just a few years ago when there was an abrupt increase of use of the term. Perhaps it is a reflection of other unfortunate recent developments in society, politics and in media (Figure 1). I believe most individuals, including laypeople, understand the idiom fake science news better than “junk science,” which presume an interest or knowledge about characteristics of good versus bad science that is not necessarily there. Therefore, I propose that ethical publishers, editors, scholars, and scientists will espouse this new powerful idiom for obvious fake, bogus, junk, and fraudulent science. I expect that no academicians will want to be associated with a negative categorization such as fake science news and will therefore perhaps refrain from supporting the predatory publishing business. An excellent initiative in this regard is the think‐check‐submit‐initiative launched in 2015 (http://thinkchecksubmit.org).

We will likely not be able to bar the predatory publishing industry, even though a major effort to curb this business has been launched in the United States by the Federal Trade Commission, who recently filed a lawsuit against one of the major actors identified as a predatory publisher (Federal Trade Commission, n.d.). The outcome of the court ruling is indeterminate at this stage. However, even if some predatory publishers choose to avoid establishing their business in USA for fear of litigation, others may operate from anywhere in the world where there is a mailbox address and a server hooked up to the Internet, that is, anywhere on our planet. Hence, our focus must principally be on educating the future generation of scholars and researchers to understand that the quantity of publications of science will never equal the peer‐recognized innovation potential and qualities of the published science.

I believe also that it would greatly benefit if mentors in academia would be more vigorous in highlighting the classic inductive method for scientific inquiry, as brilliantly rationalized by Karl Popper some 60 years ago (Popper, 1959). In short, always rationale first to create a hypothesis and then design a suitable experiment or clinical study design to disprove the hypothesis. Qualified peers will be able to identify the excellence of the hypothesis by a more or less mindful Ockham's razor test and the excellence of the experiment or study design by the provided information of its reliability and validity, as well as appropriateness, of sample size and statistical considerations for disproving the hypothesis. In contrast, most publications that today end up being printed in predatory journals simply present amassed data with no hypothesis and based on poorly described or dubious experiment or study design often accompanied by inadequate statistics. Somewhere along the road, it has escaped many that scientific research is an intellectual enterprise that goes beyond an activity based on an accrual and presentation of accumulated small or big data. Perhaps in the end, we should all reflect on our own culpability in a society where fake science news seems to be on the increase in parallel with the predatory publishing commerce.
